# Bone marrow cell transplantation efficiently repairs tendon and ligament injuries

**DOI:** 10.3389/fcell.2014.00027

**Published:** 2014-07-15

**Authors:** Taketoshi Kushida, Hirokazu Iida

**Affiliations:** Department of Orthopaedic Surgery, Kansai Medical UniversityHirakata, Japan

**Keywords:** bone marrow cell transplantation, tendon, ligament, TGF-β, fibroblast

## Introduction

Growth factors such as transforming growth factor-β (TGF-β), vascular endothelial growth factor (VEGF), insulin-like growth factor-1 (IGF-1), platelet-derived growth factor (PDGF), and basic fibroblast growth factor (bFGF) have been associated with the tendon healing process (Molloy et al., 2003; Zhang et al., 2003; Halper, 2014). Among these growth factors, TGF-β1 plays a key role during the various tissue repair stages and is secreted not only by fibroblasts differentiated from mesenchymal stem cells (MSCs), but also by several types of cells that differentiate from hematopoietic stem cells (HSCs; Sporn et al., 1986).

In this review, the role of TGF-β and MSC-derived fibroblasts in tendon and ligament tissue repair is described. Furthermore, the potential application of bone marrow cell transplantation (BMCT) as a novel method for treating tendon and ligament injuries is also discussed.

## Healing process

Human tendon healing process is classified into five phases as follows: immediate post injury phase, inflammatory phase, proliferation phase, reparative phase, and remodeling phase (Molloy et al., 2003). Tissue repair depends on both intrinsic and extrinsic processes. Intrinsic healing occurs within the tendon itself as a result of the activity of intrinsic fibroblasts and an increased intratendinous blood supply from the synovium and osseous insertion (Deie et al., 1995; Koga et al., 2007). Extrinsic factors that influence tissue repair include the availability of proteins originating from peripheral fibroblasts, plasma, inflammatory cells, and extratendinous vascular invasion (Lee et al., 1998; Zhang et al., 2003).

## Fibloblasts and collagen fibers in tissue repair

During the inflammatory phase, fibroblasts located within the tendon proliferate and circulatory fibroblasts migrate into the injured tissue site. The fibroblasts then synthesize new extracellular matrix proteins that predominantly consist of collagens and glycosaminoglycans (Molloy et al., 2003). The tendon is primarily made up of type-I collagen along with small amounts of other collagens such as type-III collagen. Type-I collagen is a mature form of collagen and is mainly responsible for the mechanical strength of the tendon. On the other hand, type-III collagen, which is thinner and more extensible than type-I collagen, is not a major collagen in normal tendon. However, type-III collagen is required during the earliest stage of tendon healing because it can rapidly cross link and stabilize the precarious repair site (Liu et al., 1995; Eriksen et al., 2002).

## Growth factors

As mentioned previously, growth factors are essential during the various stages of the tendon healing process (Molloy et al., 2003; Zhang et al., 2003; Halper, 2014). Among these growth factors, TGF-β and VEGF are important for initiating tissue repair (Molloy et al., 2003; Mienaltowski and Birk, 2014). Furthermore, TGF-β can enhance the production of VEGF (Anitua et al., 2005; Wang et al., 2005, 2008). TGF-β regulates various biological processes including cell proliferation, migration, differentiation, apoptosis, and extracellular matrix deposition (Molloy et al., 2003). In particular, TGF-β1 accelerates the proliferation and matrix synthesis of ligament fibroblasts (Schmidt et al., 1995; Marui et al., 1997; Scherping et al., 1997). In addition, TGF-β modulates proteoglycan deposition and stimulates the production of collagens by fibroblasts (Fu et al., 2005).

VEGF is also a powerful stimulator of angiogenesis and is especially important during the proliferative and remodeling phases (Molloy et al., 2003). Zhang et al. reported that the injection of exogenous VEGF in the injured site can significantly improve tensile strength of the rat Achilles tendon by stimulating growth factor production, which improves fibroblast proliferation (Zhang et al., 2003).

## Role of bone marrow cells in tissue repair

Recently, platelet-rich therapies that use a multitude of growth factors have been reported to good clinical outcomes for soft tissue injuries (Moraes et al., 2014). However, Kiapour et al stated in their review that the platelet-rich therapies have challenges associated with the short life span of these bio-active agents and the limited treatment efficacy. Furthermore. Kiapour et al stated that platelet-rich therapies required safe and reproducible systems that allow sustained delivery of growth factors to the injury site are essential (Kiapour and Murray, 2014).

Bone marrow cells (BMCs) consist of MSCs and HSCs, which can further differentiate into various cell types that secrete growth factors (Sporn et al., 1986; Baylink et al., 1993). For example, HSCs have the capacity to differentiate into growth-factor-secreting hematolymphoid cells such as lymphocytes, macrophages, granulocytes, eosinophils, erythroblasts, erythrocytes, and megakaryocytes and these cells secrete tissue repair cytokines such as TGF-β1 (Sporn et al., 1986). On the other hand, MSCs have the capacity to differentiate into several types of cells including osteocytes, chondrocytes, myotubes, stromal cells, fibroblasts, and adipocytes (Caplan, 2009; Wang et al., 2013). In particular, inflammatory cytokines such as IL-1β induce differentiation of MSCs into osteoblasts and promote mineralizing of soft tissues (Ferreira et al., 2013). MSCs themselves produce growth factors and anti-inflammatory cytokines (Le Blanc and Ringdén, 2006; Wang et al., 2008). Furthermore, MSCs differentiate into cells that participate in tissue repair and secrete growth factors and cytokines (Zantop et al., 2006; Chong et al., 2007; Ju et al., 2008). Undifferentiated MSCs produce growth factors and cytokines that promote the expansion and differentiation of HSCs and modify the response of inflammatory immune cells (Le Blanc and Ringdén, 2006).

## BMCT for treating tendon rupture

The effectiveness of MSC transplantation is due to the differentiation of MSCs into cells that participate in tissue repair and also due to the release of paracrine factors such as growth factors and cytokines by the transplanted cells (Wang et al., 2008). Previous studies show that injecting MSCs at injury sites can accelerate Achilles tendon healing in animal models (Zantop et al., 2006; Chong et al., 2007; Hou et al., 2009). Chong et al have reported that bone marrow-derived MSCs influence early tendon healing in rabbits (Chong et al., 2007). Zantop et al have reported that extracellular matrix scaffolds are repopulated by bone marrow-derived cells in a mouse model of Achilles tendon reconstruction (Zantop et al., 2006). Hou et al. have characterized the effects of *TGF-β1* and *VEGF*_165_ gene transfer on Achilles tendon healing (Hou et al., 2009).

Recently, we characterized the effects of injecting whole BMCs, which consist of both MSCs and HSCs, on tendon healing in a rat model of Achilles tendon rupture and evaluated the effects of growth factors on the early stages of healing (Okamoto et al., 2010). We showed that the levels of TGF-β and VEGF in tendons treated with BMCs were higher than those in tendons treated with MSCs. Thus, the BMC-treated group showed a rapid increase in types-I and III collagen and faster recovery of the ultimate failure load.

## BMCT for treating anterior curuciate liganent partial ruputure

Spontaneous regeneration of anterior cruciate ligament (ACL) is impeded by the lack of soft tissue around it. However, partially ruptured ACL can regenerate with growth factor administration and after cell transplantation. Intra-articular transplantation therapies using MSCs have been used for treating torn ACLs (Agung et al., 2006; Kanaya et al., 2007). Kanaya et al showed that the injected MSCs produced growth factors such as PDGF, bFGF, and TGF-β, which can activate the native ACL cells. The authors proposed that the native ACL cells may have proliferated and migrated into the injured area of the ACL. Furthermore, Agung et al have characterized the mobilization of bone marrow-derived MSCs into the injured tissues after intra-articular injection and their role in tissue regeneration (Agung et al., 2006). Taken together, these studies indicate that intra-articular transplantation therapies using MSCs have excellent outcomes for the regeneration of a transected ACL.

**Figure 1:**
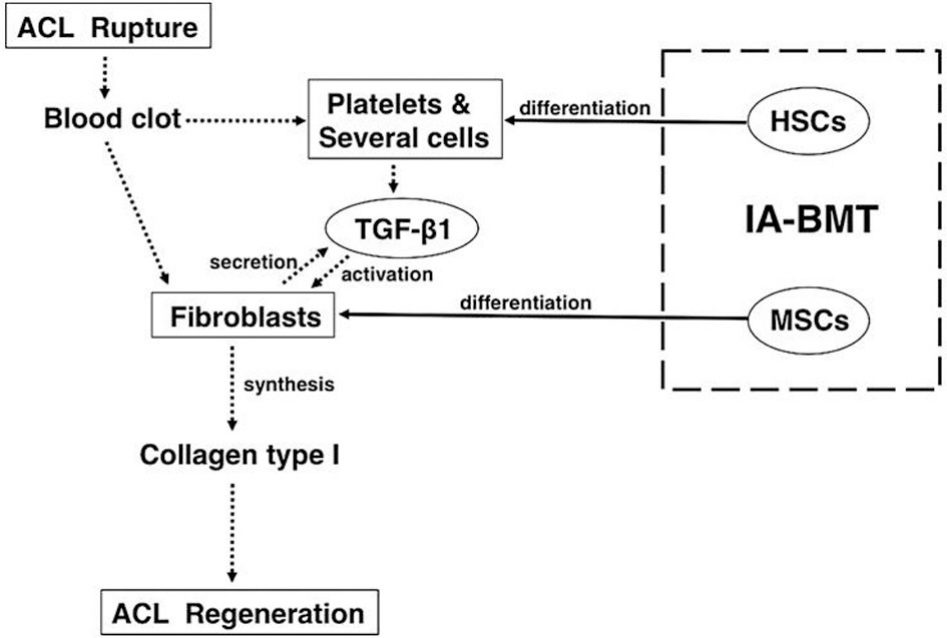
**Mechanisms underlying the treatment of anterior cruciate ligament healing by intra-articular bone marrow transplantation with fresh whole bone marrow cells.** For anterior cruciate ligament (ACL) healing, fresh whole bone marrow cells (BMCs) that are primarily comprised of mesenchymal stem cells (MSCs) and hematopoietic stem cells should be preferably used because the combination has significant advantages over MSCs only. The *dotted arrows* show the cascade of events that lead to ACL healing in general. The *solid arrows* show the effects of intra-articular bone marrow transplant with fresh whole BMCs. (This figure was taken from Oe et al., 2011).

Recently, we showed that partially transected rodent ACL can be repaired by injection of BMCs or cultured MSCs into the articular cavity at 1 week after transection (Oe et al., 2011). The rat ACLs of the BMC and MSC groups after 4 weeks of treatment appeared almost normal histologically, and the tensile strength of the ACLs of the BMC group reached near normal levels biomechanically. Furthermore, immunostaining for type-I collagen showed that the number of nuclei in the BMC group was significantly higher than that in the MSC group or the saline group. The levels of TGF-β1 in the ACL tissue and knee joint fluid in the BMC group were significantly higher than that of the saline group at 4 weeks. In conclusion, intra-articular bone marrow transplantation using fresh whole BMCs is an effective treatment for ACL partial rupture (Figure 1).

## Conclusion and futurure prospects

Stem cell therapies use several stem cell sources such as human embryonic stem cells, MSCs, BMCs, and bone marrow mononucleated cells have been reported (Chen et al., 2013). Furthermore, transplantation of the genetical transduced stem cells by “Scleraxis,” which is a transcription factor involving tendon development, have been used for effective tendon regeneration (Gulotta and Rodeo, 2011). Transplantation of MSCs that are genetically transduced with “Smad8/BMP2,” which is induced tendon formation and blocked differentiation of these cells into cartilage and bone tissues, has been reported to induce accelerated recovery of torn Achilles tendon (Pelled et al., 2012).

Few clinical trials have been performed using stem cells transplantations for treating tendon and ligament injuries in humans. Ahmad et al have reviewed 5 clinical studies that used stem cell therapy for treating tendon injuries and concluded that stem cells can have positive effect on tendon healing (Ahmad et al., 2012). In this review, we have reviewed the various processes that influence tissue repair by BMCT, which consist of both MSC and HSCs. The cells and processes involved in tendon healing are as follows: (1) fibroblasts that differentiate from MSCs secrete various growth factors and synthesize type-I collagen, (2) hematolymphoid cells that differentiate from HSCs secrete important growth factors and cytokines for extrinsic healing, and (3) microenvironments such as vascularization are improved in the damaged portion for tendon and ligament healing. Overall, BMCT may be more convenient and easily obtainable than cultured MSCs and can influence tendon and ligament repair though various healing processes.

### Conflict of interest statement

The authors declare that the research was conducted in the absence of any commercial or financial relationships that could be construed as a potential conflict of interest.
